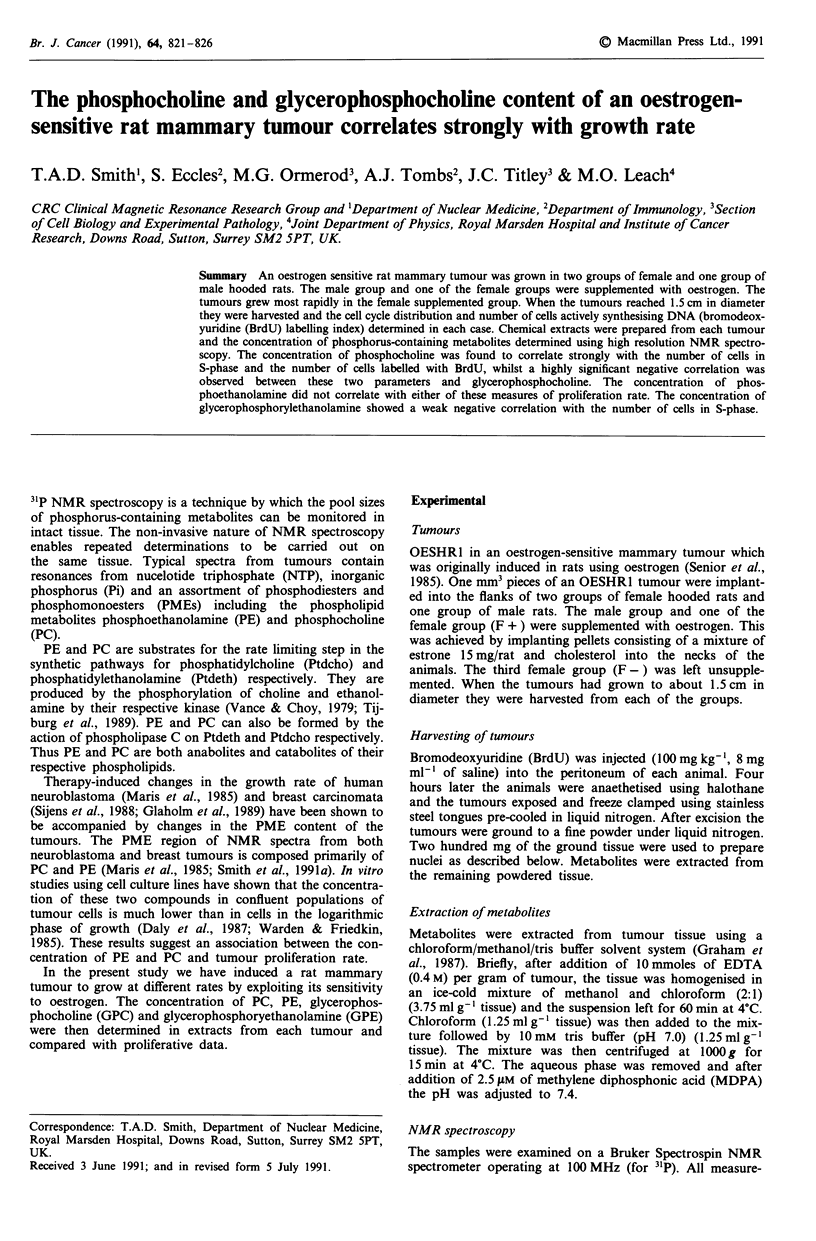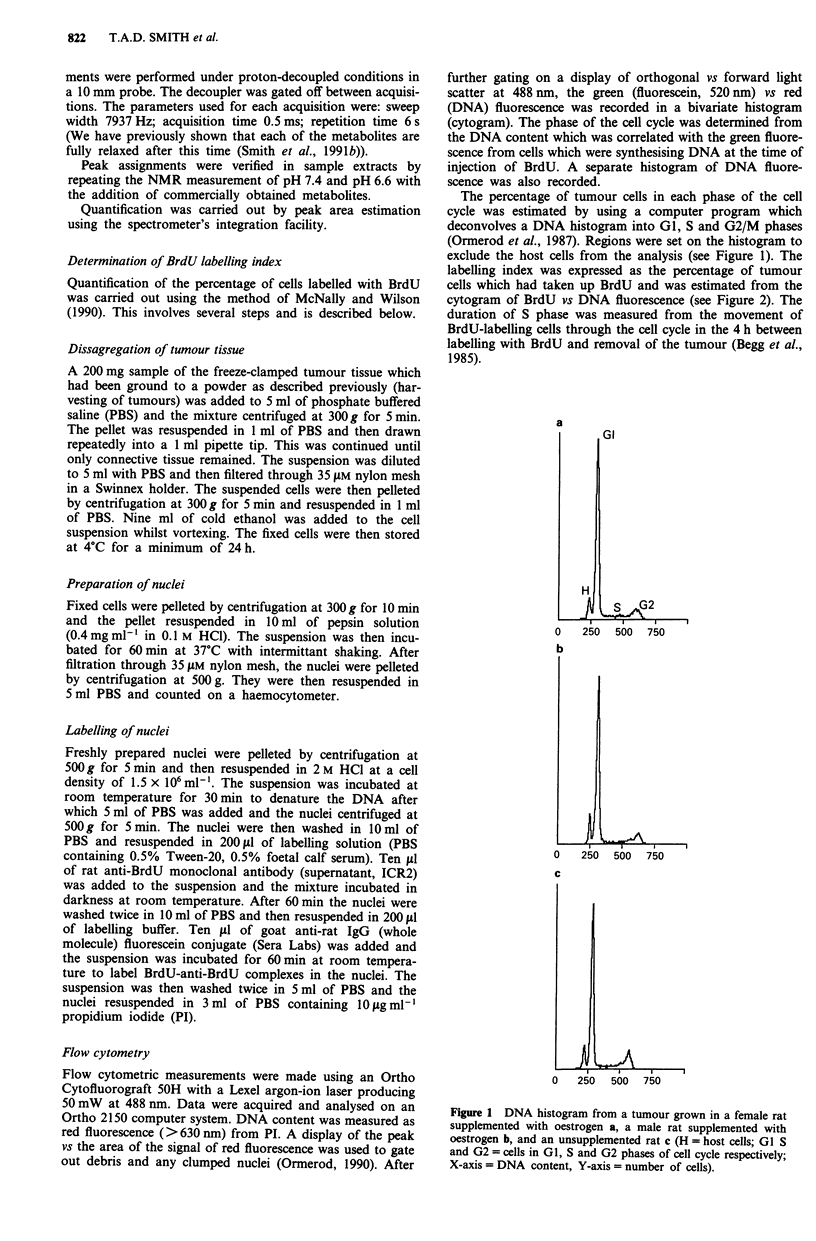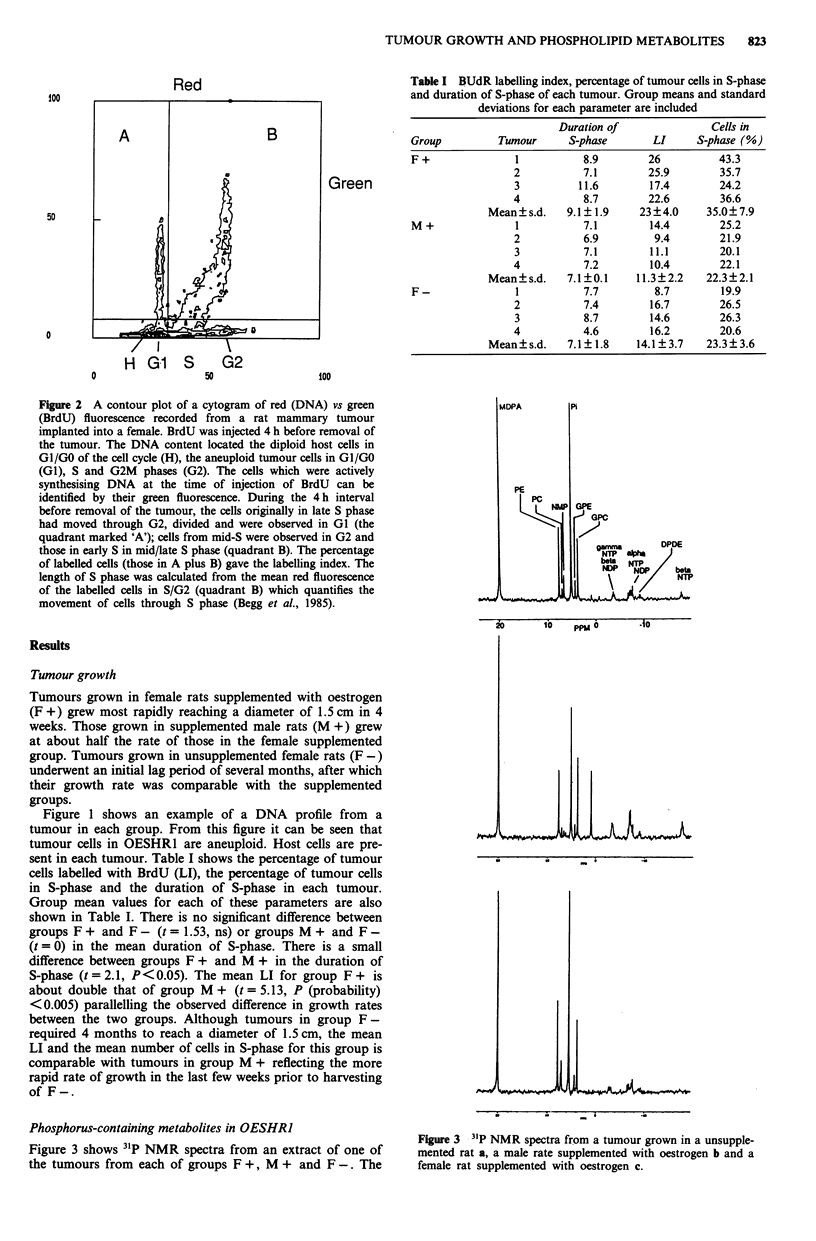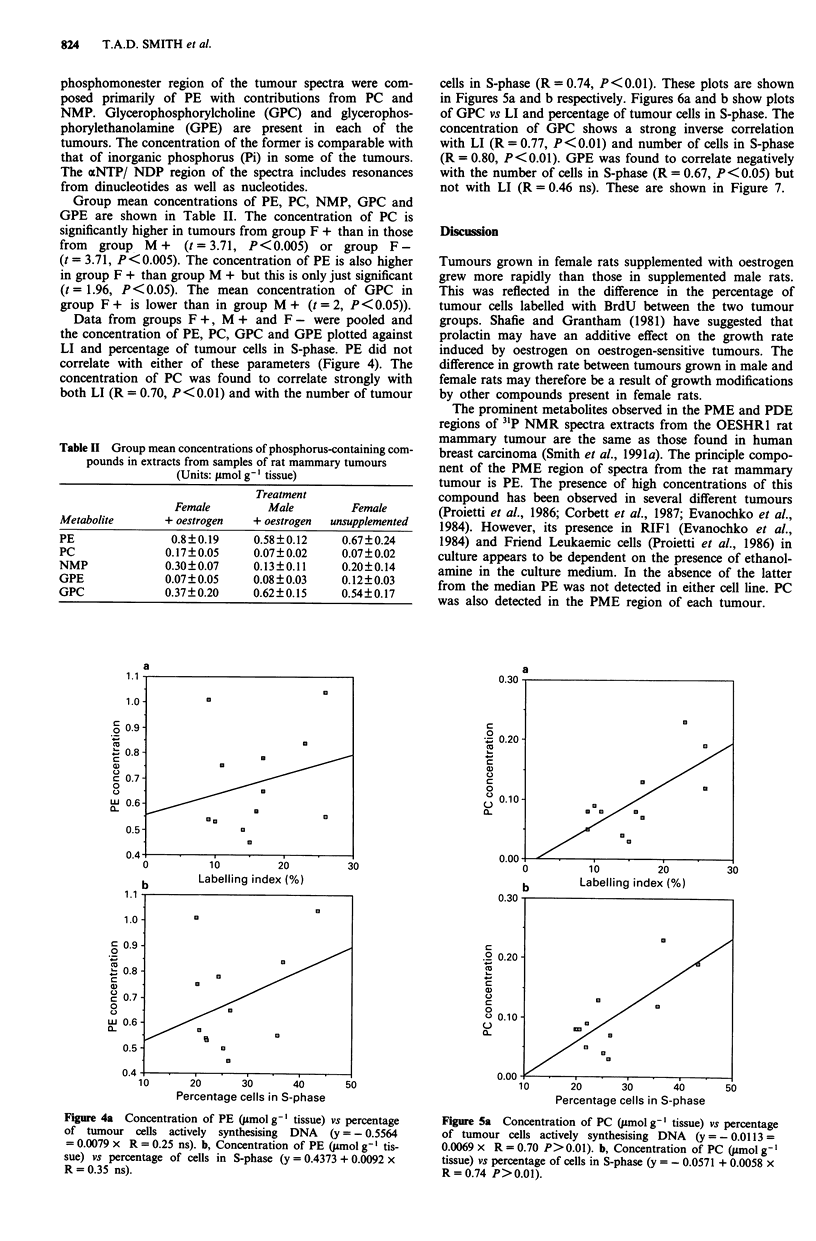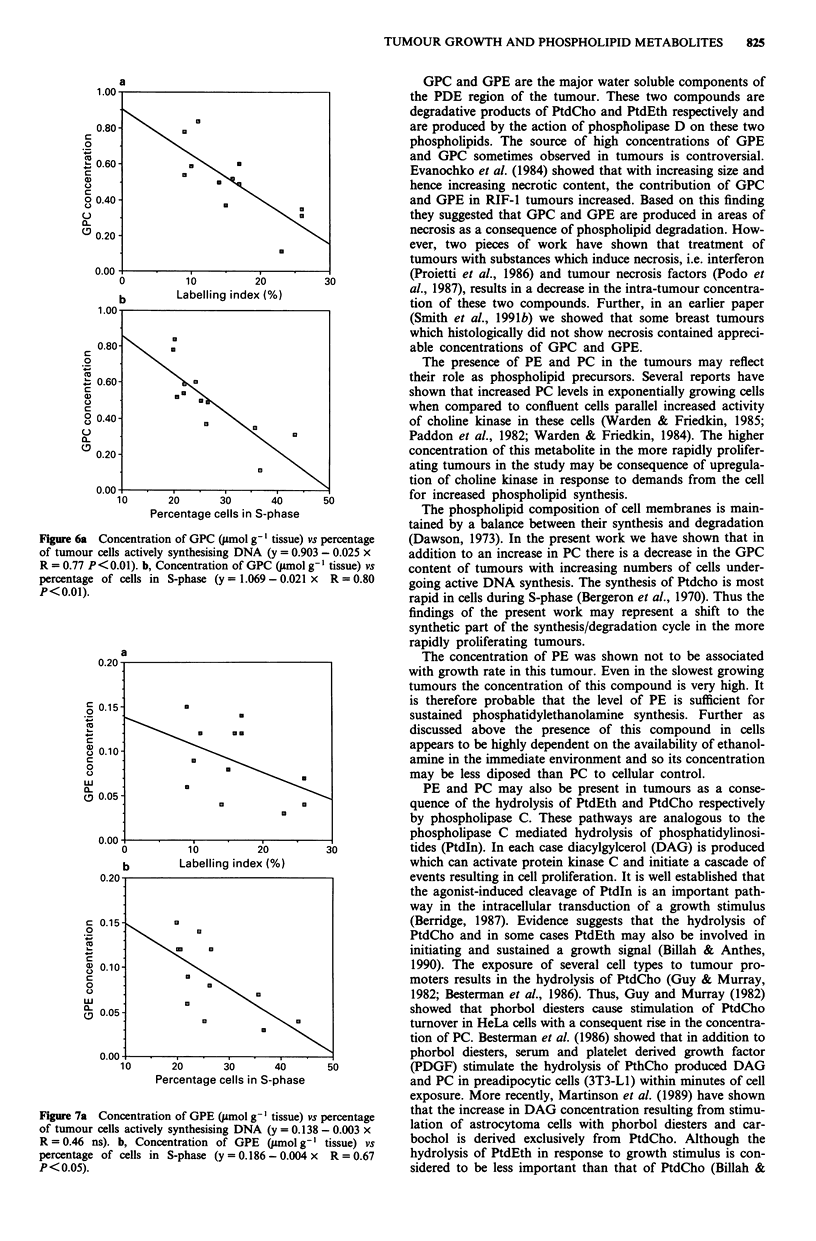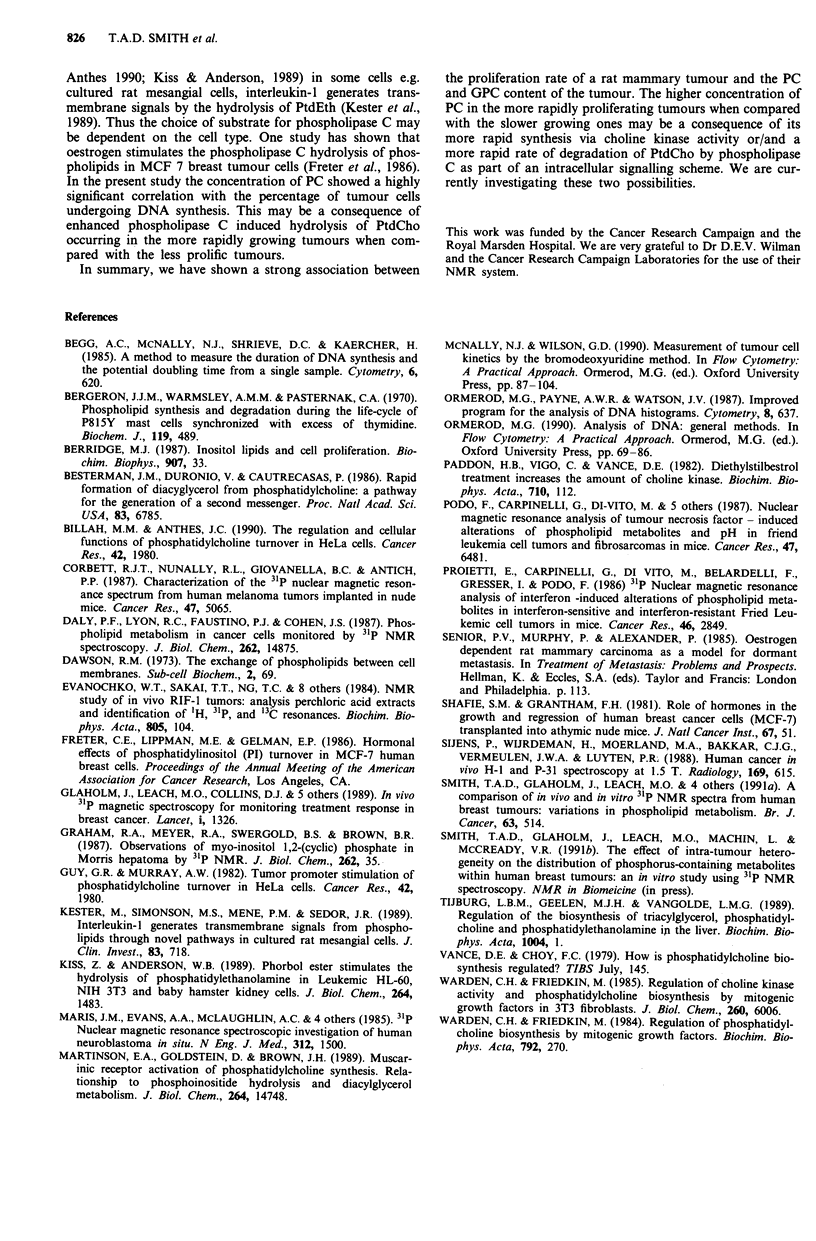# The phosphocholine and glycerophosphocholine content of an oestrogen-sensitive rat mammary tumour correlates strongly with growth rate.

**DOI:** 10.1038/bjc.1991.407

**Published:** 1991-11

**Authors:** T. A. Smith, S. Eccles, M. G. Ormerod, A. J. Tombs, J. C. Titley, M. O. Leach

**Affiliations:** Department of Nuclear Medicine, Royal Marsden Hospital, Surrey, UK.

## Abstract

An oestrogen sensitive rat mammary tumour was grown in two groups of female and one group of male hooded rats. The male group and one of the female groups were supplemented with oestrogen. The tumours grew most rapidly in the female supplemented group. When the tumours reached 1.5 cm in diameter they were harvested and the cell cycle distribution and number of cells actively synthesising DNA (bromodeoxyuridine (BrdU) labelling index) determined in each case. Chemical extracts were prepared from each tumour and the concentration of phosphorus-containing metabolites determined using high resolution NMR spectroscopy. The concentration of phosphocholine was found to correlate strongly with the number of cells in S-phase and the number of cells labelled with BrdU, whilst a highly significant negative correlation was observed between these two parameters and glycerophosphocholine. The concentration of phosphoethanolamine did not correlate with either of these measures of proliferation rate. The concentration of glycerophosphorylethanolamine showed a weak negative correlation with the number of cells in S-phase.


Br. J. Cancer (1991), 64, 821-826 © Macmillan Press Ltd., 1991

### The phosphocholine and glycerophosphocholine content of an oestrogen-sensitive rat mammary tumour correlates strongly with growth rate

T.A.D. Smith[1], S. Eccles[2], M.G. Ormerod[3], A.J. Tombs[2], J.C. Titley[3] & M.O. Leach[4]

*CRC Clinical Magnetic Resonance Research Group and [1]Department of Nuclear Medicine, [2]Department of Immunology, [3]Section of Cell Biology and Experimental Pathology, [4]Joint Department of Physics, Royal Marsden Hospital and Institute of Cancer Research, Downs Road, Sutton, Surrey SM2 5PT, UK.*

**Summary** An oestrogen sensitive rat mammary tumour was grown in two groups of female and one group of male hooded rats. The male group and one of the female groups were supplemented with oestrogen. The tumours grew most rapidly in the female supplemented group. When the tumours reached 1.5 cm in diameter they were harvested and the cell cycle distribution and number of cells actively synthesising DNA (bromodeoxyuridine (BrdU) labelling index) determined in each case. Chemical extracts were prepared from each tumour and the concentration of phosphorus-containing metabolites determined using high resolution NMR spectroscopy. The concentration of phosphocholine was found to correlate strongly with the number of cells in S-phase and the number of cells labelled with BrdU, whilst a highly significant negative correlation was observed between these two parameters and glycerophosphocholine. The concentration of phosphoethanolamine did not correlate with either of these measures of proliferation rate. The concentration of glycerophosphorylethanolamine showed a weak negative correlation with the number of cells in S-phase.

$^{31}$P NMR spectroscopy is a technique by which the pool sizes of phosphorus-containing metabolites can be monitored in intact tissue. The non-invasive nature of NMR spectroscopy enables repeated determinations to be carried out on the same tissue. Typical spectra from tumours contain resonances from nucelotide triphosphate (NTP), inorganic phosphorus (Pi) and an assortment of phosphodiesters and phosphomonoesters (PMEs) including the phospholipid metabolites phosphoethanolamine (PE) and phosphocholine (PC).

PE and PC are substrates for the rate limiting step in the synthetic pathways for phosphatidylcholine (Ptdcho) and phosphatidylethanolamine (Ptdeth) respectively. They are produced by the phosphorylation of choline and ethanolamine by their respective kinase (Vance & Choy, 1979; Tijburg *et al.*, 1989). PE and PC can also be formed by the action of phospholipase C on Ptdeth and Ptdcho respectively. Thus PE and PC are both anabolites and catabolites of their respective phospholipids.

Therapy-induced changes in the growth rate of human neuroblastoma (Maris *et al.*, 1985) and breast carcinomata (Sijens *et al.*, 1988; Glaholm *et al.*, 1989) have been shown to be accompanied by changes in the PME content of the tumours. The PME region of NMR spectra from both neuroblastoma and breast tumours is composed primarily of PC and PE (Maris *et al.*, 1985; Smith *et al.*, 1991*a*). *In vitro* studies using cell culture lines have shown that the concentration of these two compounds in confluent populations of tumour cells is much lower than in cells in the logarithmic phase of growth (Daly *et al.*, 1987; Warden & Friedkin, 1985). These results suggest an association between the concentration of PE and PC and tumour proliferation rate.

In the present study we have induced a rat mammary tumour to grow at different rates by exploiting its sensitivity to oestrogen. The concentration of PC, PE, glycerophosphocholine (GPC) and glycerophosphoryethanolamine (GPE) were then determined in extracts from each tumour and compared with proliferative data.

Correspondence: T.A.D. Smith, Department of Nuclear Medicine, Royal Marsden Hospital, Downs Road, Sutton, Surrey SM2 5PT, UK.
Received 3 June 1991; and in revised form 5 July 1991.

### Experimental

### *Tumours*

OESHR1 in an oestrogen-sensitive mammary tumour which was originally induced in rats using oestrogen (Senior *et al.*, 1985). One mm$^3$ pieces of an OESHR1 tumour were implanted into the flanks of two groups of female hooded rats and one group of male rats. The male group and one of the female group (F + ) were supplemented with oestrogen. This was achieved by implanting pellets consisting of a mixture of estrone 15 mg/rat and cholesterol into the necks of the animals. The third female group (F – ) was left unsupplemented. When the tumours had grown to about 1.5 cm in diameter they were harvested from each of the groups.

### *Harvesting of tumours*

Bromodeoxyuridine (BrdU) was injected (100 mg kg$^{-1}$, 8 mg ml$^{-1}$ of saline) into the peritoneum of each animal. Four hours later the animals were anaethetised using halothane and the tumours exposed and freeze clamped using stainless steel tongues pre-cooled in liquid nitrogen. After excision the tumours were ground to a fine powder under liquid nitrogen. Two hundred mg of the ground tissue were used to prepare nuclei as described below. Metabolites were extracted from the remaining powdered tissue.

### *Extraction of metabolites*

Metabolites were extracted from tumour tissue using a chloroform/methanol/tris buffer solvent system (Graham *et al.*, 1987). Briefly, after addition of 10 mmoles of EDTA (0.4 M) per gram of tumour, the tissue was homogenised in an ice-cold mixture of methanol and chloroform (2:1) (3.75 ml g$^{-1}$ tissue) and the suspension left for 60 min at 4°C. Chloroform (1.25 ml g$^{-1}$ tissue) was then added to the mixture followed by 10 mM tris buffer (pH 7.0) (1.25 ml g$^{-1}$ tissue). The mixture was then centrifuged at 1000 *g* for 15 min at 4°C. The aqueous phase was removed and after addition of 2.5 μM of methylene diphosphonic acid (MDPA) the pH was adjusted to 7.4.

### *NMR spectroscopy*

The samples were examined on a Bruker Spectrospin NMR spectrometer operating at 100 MHz (for $^{31}$P). All measurements were performed under proton-decoupled conditions in a 10 mm probe. The decoupler was gated off between acquisitions. The parameters used for each acquisition were: sweep width 7937 Hz; acquisition time 0.5 ms; repetition time 6 s (We have previously shown that each of the metabolites are fully relaxed after this time (Smith *et al.*, 1991*b*)).

Peak assignments were verified in sample extracts by repeating the NMR measurement of pH 7.4 and pH 6.6 with the addition of commercially obtained metabolites.

Quantification was carried out by peak area estimation using the spectrometer's integration facility.

### *Determination of BrdU labelling index*

Quantification of the percentage of cells labelled with BrdU was carried out using the method of McNally and Wilson (1990). This involves several steps and is described below.

### *Dissagregation of tumour tissue*

A 200 mg sample of the freeze-clamped tumour tissue which had been ground to a powder as described previously (harvesting of tumours) was added to 5 ml of phosphate buffered saline (PBS) and the mixture centrifuged at 300 *g* for 5 min. The pellet was resuspended in 1 ml of PBS and then drawn repeatedly into a 1 ml pipette tip. This was continued until only connective tissue remained. The suspension was diluted to 5 ml with PBS and then filtered through 35 μM nylon mesh in a Swinnex holder. The suspended cells were then pelleted by centrifugation at 300 *g* for 5 min and resuspended in 1 ml of PBS. Nine ml of cold ethanol was added to the cell suspension whilst vortexing. The fixed cells were then stored at 4°C for a minimum of 24 h.

### *Preparation of nuclei*

Fixed cells were pelleted by centrifugation at 300 *g* for 10 min and the pellet resuspended in 10 ml of pepsin solution (0.4 mg $ml^{-1}$ in 0.1 M HCl). The suspension was then incubated for 60 min at 37°C with intermittant shaking. After filtration through 35 μM nylon mesh, the nuclei were pelleted by centrifugation at 500 g. They were then resuspended in 5 ml PBS and counted on a haemocytometer.

### *Labelling of nuclei*

Freshly prepared nuclei were pelleted by centrifugation at 500 *g* for 5 min and then resuspended in 2 M HCl at a cell density of $1.5 \times 10^6$ $ml^{-1}$. The suspension was incubated at room temperature for 30 min to denature the DNA after which 5 ml of PBS was added and the nuclei centrifuged at 500 *g* for 5 min. The nuclei were then washed in 10 ml of PBS and resuspended in 200 μl of labelling solution (PBS containing 0.5% Tween-20, 0.5% foetal calf serum). Ten μl of rat anti-BrdU monoclonal antibody (supernatant, ICR2) was added to the suspension and the mixture incubated in darkness at room temperature. After 60 min the nuclei were washed twice in 10 ml of PBS and then resuspended in 200 μl of labelling buffer. Ten μl of goat anti-rat IgG (whole molecule) fluorescein conjugate (Sera Labs) was added and the suspension was incubated for 60 min at room temperature to label BrdU-anti-BrdU complexes in the nuclei. The suspension was then washed twice in 5 ml of PBS and the nuclei resuspended in 3 ml of PBS containing 10 μg $ml^{-1}$ propidium iodide (PI).

### *Flow cytometry*

Flow cytometric measurements were made using an Ortho Cytofluorograft 50H with a Lexel argon-ion laser producing 50 mW at 488 nm. Data were acquired and analysed on an Ortho 2150 computer system. DNA content was measured as red fluorescence (>630 nm) from PI. A display of the peak *vs* the area of the signal of red fluorescence was used to gate out debris and any clumped nuclei (Ormerod, 1990). After further gating on a display of orthogonal *vs* forward light scatter at 488 nm, the green (fluorescein, 520 nm) *vs* red (DNA) fluorescence was recorded in a bivariate histogram (cytogram). The phase of the cell cycle was determined from the DNA content which was correlated with the green fluorescence from cells which were synthesising DNA at the time of injection of BrdU. A separate histogram of DNA fluorescence was also recorded.

The percentage of tumour cells in each phase of the cell cycle was estimated by using a computer program which deconvolves a DNA histogram into G1, S and G2/M phases (Ormerod *et al.*, 1987). Regions were set on the histogram to exclude the host cells from the analysis (see Figure 1). The labelling index was expressed as the percentage of tumour cells which had taken up BrdU and was estimated from the cytogram of BrdU *vs* DNA fluorescence (see Figure 2). The duration of S phase was measured from the movement of BrdU-labelling cells through the cell cycle in the 4 h between labelling with BrdU and removal of the tumour (Begg *et al.*, 1985).

**Figure 1** DNA histogram from a tumour grown in a female rat supplemented with oestrogen **a**, a male rat supplemented with oestrogen **b**, and an unsupplemented rat **c** (H = host cells; G1 S and G2 = cells in G1, S and G2 phases of cell cycle respectively; X-axis = DNA content, Y-axis = number of cells).

**Figure 2** A contour plot of a cytogram of red (DNA) *vs* green (BrdU) fluorescence recorded from a rat mammary tumour implanted into a female. BrdU was injected 4 h before removal of the tumour. The DNA content located the diploid host cells in G1/G0 of the cell cycle (H), the aneuploid tumour cells in G1/G0 (G1), S and G2M phases (G2). The cells which were actively synthesising DNA at the time of injection of BrdU can be identified by their green fluorescence. During the 4 h interval before removal of the tumour, the cells originally in late S phase had moved through G2, divided and were observed in G1 (the quadrant marked 'A'); cells from mid-S were observed in G2 and those in early S in mid/late S phase (quadrant B). The percentage of labelled cells (those in A plus B) gave the labelling index. The length of S phase was calculated from the mean red fluorescence of the labelled cells in S/G2 (quadrant B) which quantifies the movement of cells through S phase (Begg *et al.*, 1985).

### Results

### *Tumour growth*

Tumours grown in female rats supplemented with oestrogen (F +) grew most rapidly reaching a diameter of 1.5 cm in 4 weeks. Those grown in supplemented male rats (M +) grew at about half the rate of those in the female supplemented group. Tumours grown in unsupplemented female rats (F −) underwent an initial lag period of several months, after which their growth rate was comparable with the supplemented groups.

Figure 1 shows an example of a DNA profile from a tumour in each group. From this figure it can be seen that tumour cells in OESHR1 are aneuploid. Host cells are present in each tumour. Table I shows the percentage of tumour cells labelled with BrdU (LI), the percentage of tumour cells in S-phase and the duration of S-phase in each tumour. Group mean values for each of these parameters are also shown in Table I. There is no significant difference between groups F + and F − ($t = 1.53$, ns) or groups M + and F − ($t = 0$) in the mean duration of S-phase. There is a small difference between groups F + and M + in the duration of S-phase ($t = 2.1$, $P < 0.05$). The mean LI for group F + is about double that of group M + ($t = 5.13$, $P$ (probability) $< 0.005$) parallelling the observed difference in growth rates between the two groups. Although tumours in group F − required 4 months to reach a diameter of 1.5 cm, the mean LI and the mean number of cells in S-phase for this group is comparable with tumours in group M + reflecting the more rapid rate of growth in the last few weeks prior to harvesting of F −.

### *Phosphorus-containing metabolites in OESHR1*

Figure 3 shows $^{31}P$ NMR spectra from an extract of one of the tumours from each of groups F +, M + and F −. The

**Table I** BUdR labelling index, percentage of tumour cells in S-phase and duration of S-phase of each tumour. Group means and standard deviations for each parameter are included

| *Group* | *Tumour* | *Duration of S-phase* | *LI* | *Cells in S-phase (%)* |
|---|---|---|---|---|
| F + | 1 | 8.9 | 26 | 43.3 |
| | 2 | 7.1 | 25.9 | 35.7 |
| | 3 | 11.6 | 17.4 | 24.2 |
| | 4 | 8.7 | 22.6 | 36.6 |
| | Mean ± s.d. | 9.1 ± 1.9 | 23 ± 4.0 | 35.0 ± 7.9 |
| M + | 1 | 7.1 | 14.4 | 25.2 |
| | 2 | 6.9 | 9.4 | 21.9 |
| | 3 | 7.1 | 11.1 | 20.1 |
| | 4 | 7.2 | 10.4 | 22.1 |
| | Mean ± s.d. | 7.1 ± 0.1 | 11.3 ± 2.2 | 22.3 ± 2.1 |
| F − | 1 | 7.7 | 8.7 | 19.9 |
| | 2 | 7.4 | 16.7 | 26.5 |
| | 3 | 8.7 | 14.6 | 26.3 |
| | 4 | 4.6 | 16.2 | 20.6 |
| | Mean ± s.d. | 7.1 ± 1.8 | 14.1 ± 3.7 | 23.3 ± 3.6 |

**Figure 3** $^{31}P$ NMR spectra from a tumour grown in a unsupplemented rat **a**, a male rate supplemented with oestrogen **b** and a female rat supplemented with oestrogen **c**.

phosphomonester region of the tumour spectra were composed primarily of PE with contributions from PC and NMP. Glycerophosphorylcholine (GPC) and glycerophosphorylethanolamine (GPE) are present in each of the tumours. The concentration of the former is comparable with that of inorganic phosphorus (Pi) in some of the tumours. The αNTP/ NDP region of the spectra includes resonances from dinucleotides as well as nucleotides.

Group mean concentrations of PE, PC, NMP, GPC and GPE are shown in Table II. The concentration of PC is significantly higher in tumours from group F + than in those from group M + ($t = 3.71$, $P < 0.005$) or group F − ($t = 3.71$, $P < 0.005$). The concentration of PE is also higher in group F + than group M + but this is only just significant ($t = 1.96$, $P < 0.05$). The mean concentration of GPC in group F + is lower than in group M + ($t = 2$, $P < 0.05$)).

Data from groups F +, M + and F − were pooled and the concentration of PE, PC, GPC and GPE plotted against LI and percentage of tumour cells in S-phase. PE did not correlate with either of these parameters (Figure 4). The concentration of PC was found to correlate strongly with both LI (R = 0.70, $P < 0.01$) and with the number of tumour cells in S-phase (R = 0.74, $P < 0.01$). These plots are shown in Figures 5a and b respectively. Figures 6a and b show plots of GPC *vs* LI and percentage of tumour cells in S-phase. The concentration of GPC shows a strong inverse correlation with LI (R = 0.77, $P < 0.01$) and number of cells in S-phase (R = 0.80, $P < 0.01$). GPE was found to correlate negatively with the number of cells in S-phase (R = 0.67, $P < 0.05$) but not with LI (R = 0.46 ns). These are shown in Figure 7.

**Table II** Group mean concentrations of phosphorus-containing compounds in extracts from samples of rat mammary tumours (Units: $\mu$mol g$^{-1}$ tissue)

| | Treatment | | |
|---|---|---|---|
| *Metabolite* | *Female + oestrogen* | *Male + oestrogen* | *Female unsupplemented* |
| PE | 0.8±0.19 | 0.58±0.12 | 0.67±0.24 |
| PC | 0.17±0.05 | 0.07±0.02 | 0.07±0.02 |
| NMP | 0.30±0.07 | 0.13±0.11 | 0.20±0.14 |
| GPE | 0.07±0.05 | 0.08±0.03 | 0.12±0.03 |
| GPC | 0.37±0.20 | 0.62±0.15 | 0.54±0.17 |

### Discussion

Tumours grown in female rats supplemented with oestrogen grew more rapidly than those in supplemented male rats. This was reflected in the difference in the percentage of tumour cells labelled with BrdU between the two tumour groups. Shafie and Grantham (1981) have suggested that prolactin may have an additive effect on the growth rate induced by oestrogen on oestrogen-sensitive tumours. The difference in growth rate between tumours grown in male and female rats may therefore be a result of growth modifications by other compounds present in female rats.

The prominent metabolites observed in the PME and PDE regions of $^{31}$P NMR spectra extracts from the OESHR1 rat mammary tumour are the same as those found in human breast carcinoma (Smith *et al.*, 1991*a*). The principle component of the PME region of spectra from the rat mammary tumour is PE. The presence of high concentrations of this compound has been observed in several different tumours (Proietti *et al.*, 1986; Corbett *et al.*, 1987; Evanochko *et al.*, 1984). However, its presence in RIF1 (Evanochko *et al.*, 1984) and Friend Leukaemic cells (Proietti *et al.*, 1986) in culture appears to be dependent on the presence of ethanolamine in the culture medium. In the absence of the latter from the median PE was not detected in either cell line. PC was also detected in the PME region of each tumour.

**Figure 4a** Concentration of PE ($\mu$mol g$^{-1}$ tissue) *vs* percentage of tumour cells actively synthesising DNA (y = − 0.5564 = 0.0079 × R = 0.25 ns). **b**, Concentration of PE ($\mu$mol g$^{-1}$ tissue) *vs* percentage of cells in S-phase (y = 0.4373 + 0.0092 × R = 0.35 ns).

**Figure 5a** Concentration of PC ($\mu$mol g$^{-1}$ tissue) *vs* percentage of tumour cells actively synthesising DNA (y = − 0.0113 = 0.0069 × R = 0.70 $P > 0.01$). **b**, Concentration of PC ($\mu$mol g$^{-1}$ tissue) *vs* percentage of cells in S-phase (y = − 0.0571 + 0.0058 × R = 0.74 $P > 0.01$).

**Figure 6a** Concentration of GPC (μmol $g^{-1}$ tissue) *vs* percentage of tumour cells actively synthesising DNA (y = 0.903 – 0.025 × R = 0.77 $P < 0.01$). **b**, Concentration of GPC (μmol $g^{-1}$ tissue) *vs* percentage of cells in S-phase (y = 1.069 – 0.021 × R = 0.80 $P < 0.01$).

**Figure 7a** Concentration of GPE (μmol $g^{-1}$ tissue) *vs* percentage of tumour cells actively synthesising DNA (y = 0.138 – 0.003 × R = 0.46 ns). **b**, Concentration of GPE (μmol $g^{-1}$ tissue) *vs* percentage of cells in S-phase (y = 0.186 – 0.004 × R = 0.67 $P < 0.05$).

GPC and GPE are the major water soluble components of the PDE region of the tumour. These two compounds are degradative products of PtdCho and PtdEth respectively and are produced by the action of phospholipase D on these two phospholipids. The source of high concentrations of GPE and GPC sometimes observed in tumours is controversial. Evanochko *et al.* (1984) showed that with increasing size and hence increasing necrotic content, the contribution of GPC and GPE in RIF-1 tumours increased. Based on this finding they suggested that GPC and GPE are produced in areas of necrosis as a consequence of phospholipid degradation. However, two pieces of work have shown that treatment of tumours with substances which induce necrosis, i.e. interferon (Proietti *et al.*, 1986) and tumour necrosis factors (Podo *et al.*, 1987), results in a decrease in the intra-tumour concentration of these two compounds. Further, in an earlier paper (Smith *et al.*, 1991*b*) we showed that some breast tumours which histologically did not show necrosis contained appreciable concentrations of GPC and GPE.

The presence of PE and PC in the tumours may reflect their role as phospholipid precursors. Several reports have shown that increased PC levels in exponentially growing cells when compared to confluent cells parallel increased activity of choline kinase in these cells (Warden & Friedkin, 1985; Paddon *et al.*, 1982; Warden & Friedkin, 1984). The higher concentration of this metabolite in the more rapidly proliferating tumours in the study may be consequence of upregulation of choline kinase in response to demands from the cell for increased phospholipid synthesis.

The phospholipid composition of cell membranes is maintained by a balance between their synthesis and degradation (Dawson, 1973). In the present work we have shown that in addition to an increase in PC there is a decrease in the GPC content of tumours with increasing numbers of cells undergoing active DNA synthesis. The synthesis of Ptdcho is most rapid in cells during S-phase (Bergeron *et al.*, 1970). Thus the findings of the present work may represent a shift to the synthetic part of the synthesis/degradation cycle in the more rapidly proliferating tumours.

The concentration of PE was shown not to be associated with growth rate in this tumour. Even in the slowest growing tumours the concentration of this compound is very high. It is therefore probable that the level of PE is sufficient for sustained phosphatidylethanolamine synthesis. Further as discussed above the presence of this compound in cells appears to be highly dependent on the availability of ethanolamine in the immediate environment and so its concentration may be less diposed than PC to cellular control.

PE and PC may also be present in tumours as a consequence of the hydrolysis of PtdEth and PtdCho respectively by phospholipase C. These pathways are analogous to the phospholipase C mediated hydrolysis of phosphatidylinositides (PtdIn). In each case diacylgylcerol (DAG) is produced which can activate protein kinase C and initiate a cascade of events resulting in cell proliferation. It is well established that the agonist-induced cleavage of PtdIn is an important pathway in the intracellular transduction of a growth stimulus (Berridge, 1987). Evidence suggests that the hydrolysis of PtdCho and in some cases PtdEth may also be involved in initiating and sustained a growth signal (Billah & Anthes, 1990). The exposure of several cell types to tumour promoters results in the hydrolysis of PtdCho (Guy & Murray, 1982; Besterman *et al.*, 1986). Thus, Guy and Murray (1982) showed that phorbol diesters cause stimulation of PtdCho turnover in HeLa cells with a consequent rise in the concentration of PC. Besterman *et al.* (1986) showed that in addition to phorbol diesters, serum and platelet derived growth factor (PDGF) stimulate the hydrolysis of PthCho produced DAG and PC in preadipocytic cells (3T3-L1) within minutes of cell exposure. More recently, Martinson *et al.* (1989) have shown that the increase in DAG concentration resulting from stimulation of astrocytoma cells with phorbol diesters and carbochol is derived exclusively from PtdCho. Although the hydrolysis of PtdEth in response to growth stimulus is considered to be less important than that of PtdCho (Billah &

Anthes 1990; Kiss & Anderson, 1989) in some cells e.g. cultured rat mesangial cells, interleukin-1 generates transmembrane signals by the hydrolysis of PtdEth (Kester et al., 1989). Thus the choice of substrate for phospholipase C may be dependent on the cell type. One study has shown that oestrogen stimulates the phospholipase C hydrolysis of phospholipids in MCF 7 breast tumour cells (Freter et al., 1986). In the present study the concentration of PC showed a highly significant correlation with the percentage of tumour cells undergoing DNA synthesis. This may be a consequence of enhanced phospholipase C induced hydrolysis of PtdCho occurring in the more rapidly growing tumours when compared with the less prolific tumours.

In summary, we have shown a strong association between the proliferation rate of a rat mammary tumour and the PC and GPC content of the tumour. The higher concentration of PC in the more rapidly proliferating tumours when compared with the slower growing ones may be a consequence of its more rapid synthesis via choline kinase activity or/and a more rapid rate of degradation of PtdCho by phospholipase C as part of an intracellular signalling scheme. We are currently investigating these two possibilities.

This work was funded by the Cancer Research Campaign and the Royal Marsden Hospital. We are very grateful to Dr D.E.V. Wilman and the Cancer Research Campaign Laboratories for the use of their NMR system.

### References

BEGG, A.C., MCNALLY, N.J., SHRIEVE, D.C. & KAERCHER, H. (1985). A method to measure the duration of DNA synthesis and the potential doubling time from a single sample. *Cytometry*, **6**, 620.

BERGERON, J.J.M., WARMSLEY, A.M.M. & PASTERNAK, C.A. (1970). Phospholipid synthesis and degradation during the life-cycle of P815Y mast cells synchronized with excess of thymidine. *Biochem. J.*, **119**, 489.

BERRIDGE, M.J. (1987). Inositol lipids and cell proliferation. *Biochim. Biophys.*, **907**, 33.

BESTERMAN, J.M., DURONIO, V. & CAUTRECASAS, P. (1986). Rapid formation of diacyglycerol from phosphatidylcholine: a pathway for the generation of a second messenger. *Proc. Natl Acad. Sci. USA*, **83**, 6785.

BILLAH, M.M. & ANTHES, J.C. (1990). The regulation and cellular functions of phosphatidylcholine turnover in HeLa cells. *Cancer Res.*, **42**, 1980.

CORBETT, R.J.T., NUNALLY, R.L., GIOVANELLA, B.C. & ANTICH, P.P. (1987). Characterization of the $^{31}$P nuclear magnetic resonance spectrum from human melanoma tumors implanted in nude mice. *Cancer Res.*, **47**, 5065.

DALY, P.F., LYON, R.C., FAUSTINO, P.J. & COHEN, J.S. (1987). Phospholipid metabolism in cancer cells monitored by $^{31}$P NMR spectroscopy. *J. Biol. Chem.*, **262**, 14875.

DAWSON, R.M. (1973). The exchange of phospholipids between cell membranes. *Sub-cell Biochem.*, **2**, 69.

EVANOCHKO, W.T., SAKAI, T.T., NG, T.C. & 8 others (1984). NMR study of in vivo RIF-1 tumors: analysis perchloric acid extracts and identification of $^{1}$H, $^{31}$P, and $^{13}$C resonances. *Biochim. Biophys. Acta.*, **805**, 104.

FRETER, C.E., LIPPMAN, M.E. & GELMAN, E.P. (1986). Hormonal effects of phosphatidylinositol (PI) turnover in MCF-7 human breast cells. *Proceedings of the Annual Meeting of the American Association for Cancer Research*, Los Angeles, CA.

GLAHOLM, J., LEACH, M.O., COLLINS, D.J. & 5 others (1989). *In vivo* $^{31}$P magnetic spectroscopy for monitoring treatment response in breast cancer. *Lancet*, **i**, 1326.

GRAHAM, R.A., MEYER, R.A., SWERGOLD, B.S. & BROWN, B.R. (1987). Observations of myo-inositol 1,2-(cyclic) phosphate in Morris hepatoma by $^{31}$P NMR. *J. Biol. Chem.*, **262**, 35.

GUY, G.R. & MURRAY, A.W. (1982). Tumor promoter stimulation of phosphatidylcholine turnover in HeLa cells. *Cancer Res.*, **42**, 1980.

KESTER, M., SIMONSON, M.S., MENE, P.M. & SEDOR, J.R. (1989). Interleukin-1 generates transmembrane signals from phospholipids through novel pathways in cultured rat mesangial cells. *J. Clin. Invest.*, **83**, 718.

KISS, Z. & ANDERSON, W.B. (1989). Phorbol ester stimulates the hydrolysis of phosphatidylethanolamine in Leukemic HL-60, NIH 3T3 and baby hamster kidney cells. *J. Biol. Chem.*, **264**, 1483.

MARIS, J.M., EVANS, A.A., MCLAUGHLIN, A.C. & 4 others (1985). $^{31}$P Nuclear magnetic resonance spectroscopic investigation of human neuroblastoma *in situ*. *N Eng. J. Med.*, **312**, 1500.

MARTINSON, E.A., GOLDSTEIN, D. & BROWN, J.H. (1989). Muscarinic receptor activation of phosphatidylcholine synthesis. Relationship to phosphoinositide hydrolysis and diacylglycerol metabolism. *J. Biol. Chem.*, **264**, 14748.

MCNALLY, N.J. & WILSON, G.D. (1990). Measurement of tumour cell kinetics by the bromodeoxyuridine method. In *Flow Cytometry: A Practical Approach*. Ormerod, M.G. (ed.). Oxford University Press, pp. 87–104.

ORMEROD, M.G., PAYNE, A.W.R. & WATSON, J.V. (1987). Improved program for the analysis of DNA histograms. *Cytometry*, **8**, 637.

ORMEROD, M.G. (1990). Analysis of DNA: general methods. In *Flow Cytometry: A Practical Approach*. Ormerod, M.G. (ed.). Oxford University Press, pp. 69–86.

PADDON, H.B., VIGO, C. & VANCE, D.E. (1982). Diethylstilbestrol treatment increases the amount of choline kinase. *Biochim. Biophys. Acta.*, **710**, 112.

PODO, F., CARPINELLI, G., DI-VITO, M. & 5 others (1987). Nuclear magnetic resonance analysis of tumour necrosis factor – induced alterations of phospholipid metabolites and pH in friend leukemia cell tumors and fibrosarcomas in mice. *Cancer Res.*, **47**, 6481.

PROIETTI, E., CARPINELLI, G., DI VITO, M., BELARDELLI, F., GRESSER, I. & PODO, F. (1986) $^{31}$P Nuclear magnetic resonance analysis of interferon -induced alterations of phospholipid metabolites in interferon-sensitive and interferon-resistant Fried Leukemic cell tumors in mice. *Cancer Res.*, **46**, 2849.

SENIOR, P.V., MURPHY, P. & ALEXANDER, P. (1985). Oestrogen dependent rat mammary carcinoma as a model for dormant metastasis. In *Treatment of Metastasis: Problems and Prospects*. Hellman, K. & Eccles, S.A. (eds). Taylor and Francis: London and Philadelphia. p. 113.

SHAFIE, S.M. & GRANTHAM, F.H. (1981). Role of hormones in the growth and regression of human breast cancer cells (MCF-7) transplanted into athymic nude mice. *J. Natl Cancer Inst.*, **67**, 51.

SIJENS, P., WIJRDEMAN, H., MOERLAND, M.A., BAKKAR, C.J.G., VERMEULEN, J.W.A. & LUYTEN, P.R. (1988). Human cancer *in vivo* H-1 and P-31 spectroscopy at 1.5 T. *Radiology*, **169**, 615.

SMITH, T.A.D., GLAHOLM, J., LEACH, M.O. & 4 others (1991*a*). A comparison of *in vivo* and *in vitro* $^{31}$P NMR spectra from human breast tumours: variations in phospholipid metabolism. *Br. J. Cancer*, **63**, 514.

SMITH, T.A.D., GLAHOLM, J., LEACH, M.O., MACHIN, L. & MCCREADY, V.R. (1991*b*). The effect of intra-tumour heterogeneity on the distribution of phosphorus-containing metabolites within human breast tumours: an *in vitro* study using $^{31}$P NMR spectroscopy. *NMR in Biomeicine* (in press).

TIJBURG, L.B.M., GEELEN, M.J.H. & VANGOLDE, L.M.G. (1989). Regulation of the biosynthesis of triacylglycerol, phosphatidylcholine and phosphatidylethanolamine in the liver. *Biochim. Biophys. Acta*, **1004**, 1.

VANCE, D.E. & CHOY, F.C. (1979). How is phosphatidylcholine biosynthesis regulated? *TIBS* July, 145.

WARDEN, C.H. & FRIEDKIN, M. (1985). Regulation of choline kinase activity and phosphatidylcholine biosynthesis by mitogenic growth factors in 3T3 fibroblasts. *J. Biol. Chem.*, **260**, 6006.

WARDEN, C.H. & FRIEDKIN, M. (1984). Regulation of phosphatidylcholine biosynthesis by mitogenic growth factors. *Biochim. Biophys. Acta*, **792**, 270.